# Let’s Get Physical! A Comprehensive Review of Pre- and Post-Surgical Interventions Targeting Physical Activity to Improve Pain and Functional Outcomes in Spine Surgery Patients

**DOI:** 10.3390/jcm12072608

**Published:** 2023-03-30

**Authors:** Bethany D. Pester, Jihee Yoon, Jolin B. Yamin, Lauren Papianou, Robert R. Edwards, Samantha M. Meints

**Affiliations:** 1 Department of Anesthesiology, Perioperative and Pain Medicine, Brigham and Women’s Hospital, Chestnut Hill, MA 02467, USA; 2 Harvard Medical School, Boston, MA 02115, USA; 3 Department of Emergency Medicine, Beth Israel Deaconess Medical Center, Boston, MA 02215, USA

**Keywords:** spine surgery, rehabilitation, physiotherapy, psychotherapy, physical activity, pain outcomes

## Abstract

The goal of this comprehensive review was to synthesize the recent literature on the efficacy of perioperative interventions targeting physical activity to improve pain and functional outcomes in spine surgery patients. Overall, research in this area does not yet permit definitive conclusions. Some evidence suggests that post-surgical interventions may yield more robust long-term outcomes than preoperative interventions, including large effect sizes for disability reduction, although there are no studies directly comparing these surgical approaches. Integrated treatment approaches that include psychosocial intervention components may supplement exercise programs by addressing fear avoidance behaviors that interfere with engagement in activity, thereby maximizing the short- and long-term benefits of exercise. Efforts should be made to test brief, efficient programs that maximize accessibility for surgical patients. Future work in this area should include both subjective and objective indices of physical activity as well as investigating both acute postoperative outcomes and long-term outcomes.

## 1. Introduction

Rates of spinal surgery are rapidly increasing [1,2]. Although spine surgery is generally considered a safe and effective treatment, approximately 20% of patients experience continued or recurrent chronic post-surgical pain [3], and 15–35% report no significant improvement in physical function after surgery [4,5]. Opioid medications are often used to treat pain following surgery [6,7,8,9], but notably, up to 80% of spinal surgery patients become chronic opioid users—the highest percentage of any surgical population [9,10]. Furthermore, long-term opioid use after spinal surgery is associated with depression, additional surgeries, and extended leave from work [11]. Given the risks associated with opioids and poor outcomes among many spinal surgery patients, there is a need for non-pharmacological approaches to safely enhance outcomes and prevent long-term sequelae of chronic pain after surgery.

One potential behavioral target to enhance surgical outcomes is physical activity. Physical activity, such as walking, is, importantly, associated with recovery of function and less pain-related disability after spinal surgery [12,13,14,15,16] and is generally shown to benefit people with chronic pain [17]. In a cross-sectional study in patients with chronic pain, those who reported more frequent walking and engagement in moderate activity also reported better scores on measures of patient functioning [18]. Despite the potential benefits of physical activity on pain outcomes, movement is often avoided by those with persistent pain due to worries of injury and/or exacerbating pain. Paradoxically, the avoidance of activity due to pain or fear often results in increased distress, disability, and even pain itself [8,19,20]. The fear–avoidance cycle may be particularly relevant to surgical populations who are often given variable advice from providers regarding walking and physical activity [21]. Anecdotally, surgical patients have expressed uncertainty about the amount and type of activity they should engage in pre- and post-operatively, along with fears of overdoing physical activity. Guidance is needed for both patients and providers based on the most up-to-date empirical support.

**Figure 1:**
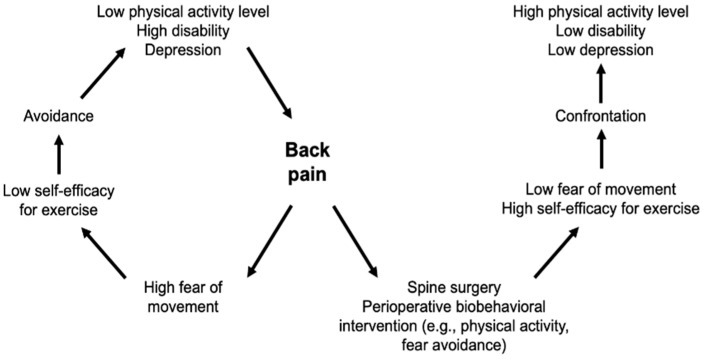
Modified fear–avoidance model for spine surgery patients.

Given the documented benefits of physical activity for spine surgery patients, along with patients’ potential fear of movement, interventions aimed at promoting physical activity may be particularly beneficial and could be incorporated into perioperative treatment (see Figure 1). To our knowledge, there are no current reviews of the evidence for both pre- and post-surgical interventions to promote physical activity, and thereby, pain outcomes, in spine surgery. Such interventions may range from exercise programs to multidisciplinary rehabilitation. The purpose of this comprehensive review was to synthesize the recent literature on the efficacy of pre- and post-surgical interventions targeting physical activity to improve pain and functional outcomes in spine surgery patients. We reviewed recent clinical trials (2015–2022) that tested the effects of either prehabilitation or post-operative intervention (e.g., exercise programs, integrated treatment) on physical activity and pain-related outcomes in all types of spine surgery patients (e.g., lumbar, cervical, degenerative).

## 2. Pre-Surgical Interventions to Promote Physical Activity

Higher levels of preoperative physical activity and fitness are associated with faster recovery and shorter hospital stays following spinal surgery [22,23]. However, patients awaiting such surgery often suffer from debilitating pain that interferes with daily activities, leading to a relatively sedentary lifestyle [24]. Given the relationship between pre-surgical physical activity and greater post-surgical outcomes, exercise-based prehabilitation has garnered attention.

### 2.1. Exercise Programs

Exercise-based prehabilitation aims to enhance patients’ physiological fitness in preparation for a surgical procedure to minimize physical deterioration and promote faster recovery [25]. Studies examining the efficacy of prehabilitation in spine surgery patients have yielded mixed results. Though patients who engage in prehabilitation generally show improvements in physical capacities preoperatively, there is inconsistent evidence of enhanced outcomes postoperatively. For example, a randomized trial showed that spinal surgery patients who engaged in pre-surgery physiotherapy twice a week for 9 weeks (i.e., a supervised exercise program and behavioral approach to reduce fear avoidance and increase activity level) had better preoperative outcomes than a waitlist control group, including reduced self-reported back pain and symptoms of psychological distress, as well as an enhanced quality of life, self-efficacy, and physical activity level [26]. However, only improvements in physical activity were maintained following the surgical procedure. A secondary analysis from this trial examined self-report and objective measures of physical function [27] and found that pre-surgery physiotherapy led to statistically significant, albeit small, improvements in walking ability and lower extremity strength prior to surgery, compared with the usual care, and that postoperative physical activity levels were, in part, explained by pre-surgical levels of physical activity. Another randomized trial comparing a 6-week supervised exercise-based prehabilitation program to usual care found that spine surgery patients who received prehabilitation showed preoperative improvements in both self-reported clinical and objective physical outcomes (e.g., leg pain intensity, lumbar spinal stenosis-related disability, maximum lumbar strength in flexion, low back extensor muscles endurance, total ambulation time, and sit to stand) [28]. Nevertheless, only improvements in low back-related disability were sustained postoperatively.

Other studies using a more targeted approach offer stronger support for prehabilitation for spine surgery patients. A recent pilot study [29], for example, enrolled only high-risk patients (i.e., deconditioned patients with poor physical capacity) undergoing lumbar spinal fusion, and compared those who received preoperative functional high-intensity interval training (f-HIIT) with matched controls who received usual care. Importantly, the eight supervised f-HIIT sessions were individualized to patients’ needs and were functional and community-based so that patients could incorporate the exercises into their daily life. Findings from this pilot trial demonstrated that preoperative community-based f-HIIT is feasible and safe for high-risk patients and may shorten the time to functional recovery after spine surgery.

Overall, the current evidence for exercise-based prehabilitation for spine surgery patients is limited. However, the more robust findings for other major joint surgeries such as total knee replacement suggest that additional work in this area is likely warranted (e.g., recent meta-analyses indicate that preoperative exercise reduces pain and disability after knee replacement surgery) [30,31]. The extant spine surgery literature suggests that a targeted approach with high-risk (e.g., deconditioned) patients may be most appropriate. It is also possible that exercise-based prehabilitation alone may be insufficient to effectively enhance postoperative outcomes and complementary approaches may be needed.

### 2.2. Integrated Approaches

Recognizing the importance of cognitive-behavioral factors in promoting physical activity and pain management, researchers have begun to test preoperative integrated approaches for spine surgery patients, including psychological intervention. Broadly, psychological interventions, such as cognitive behavioral therapy (CBT) and motivational interviewing (MI), have long been used to help individuals improve their physical activity [32,33,34]. Though such approaches seem promising, a recent systematic review and meta-analysis of 15 studies of prehabilitation interventions for lumbar spine surgery indicated no effect of pre-surgical CBT across any post-surgical outcomes including self-reported physical function at short-, medium-, and long-term follow-up, objective functional testing (e.g., timed-up-and-go test, 6 min walk task), back or leg pain, health-related quality of life, depression, anxiety, hospital length of stay, or analgesic use [35]. Despite these null findings, the heterogeneity among included studies precludes drawing definitive conclusions. Overall, however, unimodal interventions (e.g., exercise, CBT) may not be adequate in creating lasting changes among surgical patients. Other approaches may be warranted, including multi-component treatments delivered by interdisciplinary teams, along with the inclusion of post-surgical (in addition to pre-surgical) interventions.

## 3. Post-Surgical Interventions to Promote Physical Activity

Regular activity post-surgery may facilitate mobility and restore back strength. The extent to which patients engage in postoperative physical activity, including but not limited to prescribed physical therapy, and the types of exercises they engage in can vary considerably. On average, patients have been shown to walk for less than an hour a day over the week after lumbar surgery [36]. During this acute postoperative period, a lower step count has been linked to a longer time to achieve independent mobility and a longer hospital admission [36]. Thus, researchers have begun testing postoperative interventions to enhance activity and optimize surgical outcomes.

### 3.1. Exercise Programs

Postoperative exercise programs may include walking, stretching, and strengthening movements [37] and have been shown to reduce short-term pain and disability [38,39]. A recent systematic review of exercise interventions following lumbar decompression surgery identified 14 studies addressing a variety of exercise-based interventions, including strengthening, stabilization, and aerobic training [40]. Overall, these programs produced large short-term benefits (relative to usual care and education) on disability scores up to 3 months after surgery (Standardized Effect Size estimate of −0.87 for the reduction in disability) and moderate benefits on pain reduction over the same time frame (Standardized Effect Size estimate of −0.35 for the reduction in pain). Overall, early exercise-based intervention after lumbar spine surgery appears beneficial in improving pain and functional outcomes, though there is no consensus on the optimal time to initiate such programs [38,41,42,43,44]. Moreover, the evidence for long-term benefits awaits additional trials with longer-term follow-up periods.

Several recent studies focusing on the early initiation of rehabilitation after spinal surgery have yielded mixed long-term results. For instance, Kernc and colleagues [45] found that lumbar fusion patients who received physiotherapy twice a week for 9 weeks starting at 3 weeks postoperatively (e.g., strength training focused on lumbopelvic stabilization muscles) had significantly better walking speed and isometric lateral flexion strength at a 3-month follow-up compared with those receiving a standard postoperative protocol where no exercises were performed at that stage of rehabilitation; though the benefits had faded by 18 months after surgery. Meanwhile, Zhang and colleagues [46] found that early physiotherapy, implemented the day after microdiscectomy, led to better physical and functional outcomes at 12 months post-surgery (e.g., lumbar curvature, lumbar lordosis, sacral inclination angle, function, quality of life). In another randomized trial of patients who had undergone lumbar disc surgery, participants who received 2–6, 10–30 min sessions of postoperative physical therapy starting on day 1 post-surgery had less leg pain and better physical performance at a 12-month follow-up than those who had received education only [47]. In contrast, an RCT by Oosterhuis et al. [48] found that early rehabilitation did not improve pain, functional status, or mental health at a 26-week follow-up, compared with a no-exercise control group.

Overall, there is significant evidence supporting the short-term benefits of postoperative exercise programs among patients undergoing spinal surgery, with large effect sizes on measures of patient-reported disability up to 3 months post-surgery. Evidence for longer-term benefits is less definitive, but very early postoperative physiotherapy (e.g., starting on post-operative day 1) in particular, appears to be safe and may confer potentially long-term benefits.

### 3.2. Integrated Approaches

Integrated approaches such as multidisciplinary rehabilitation have also been tested with patients post-spine surgery. Many postoperative integrated approaches combine exercise with psychologically oriented content, such as CBT or psychoeducation. There appears to be great variability in the types of integrated interventions tested in this population in terms of duration, structure, and setting. Greenwood and colleagues [49] evaluated a multidisciplinary rehabilitation program that comprised ten 90 min consecutive weekly group rehabilitation sessions consisting of psychoeducation, supervised exercise, and group discussion. Other approaches involved fewer and/or shorter sessions and home-based delivery. For example, Abbott et al. [50] tested the effects of a home program and three 90 min physiotherapy sessions, and Archer et al. [51] studied a cognitive behavioral-based physical therapy program consisting of six 30 min weekly sessions with a physical therapist. Monticone and colleagues [52] tested a highly time-intensive integrated intervention consisting of eight 60 min sessions of CBT followed by twenty 90 min sessions of exercise training, totaling approximately 38 h of intervention time. A consistent theme across studies of integrated programs is a focus on addressing maladaptive pain cognitions (e.g., catastrophizing) and fear of movement (i.e., kinesiophobia) in conjunction with physical exercise components.

Overall, postoperative integrated approaches are shown to improve self-reported disability and fear avoidance behavior for spine surgery patients compared to usual post-surgical care; however, the effects of such programs on pain severity and psychological symptoms are less consistent [53]. A recent meta-analysis on postoperative integrated approaches for spine surgery patients indicated that CBT plus exercise interventions were not superior to exercise therapy alone on long-term pain and quality of life; however, CBT plus exercise yielded better results on long-term, self-reported disability and fear avoidance behavior [40]. Overall, there is a lack of high-quality research on integrated or multidisciplinary post-surgical interventions and the currently used interventions are markedly different across studies.

## 4. Conclusions

The goal of this comprehensive review was to synthesize the recent literature on the efficacy of pre- and post-operative interventions targeting physical activity to improve pain and functional outcomes in spine surgery patients. While methodologies and conclusions show substantial variability across studies, recent systematic reviews report moderate effect sizes for pain reduction and large effect sizes for improvement in disability when evaluating the benefits of activity- and exercise-based interventions delivered postoperatively for patients undergoing spine surgery. Overall, the variation in findings across studies may be partly attributable to differences in how physical activity and post-surgical pain outcomes are operationalized and measured, including self-report measures (e.g., the Oswestry Disability Index, the Numeric Rating Scale, the Patient-Reported Outcomes Measurement Information System) and objective measures (e.g., accelerometers, physical performance testing). Associations between subjective self-report measures of activity and objective indices of activity are often quite modest in studies of patients with pain [54]. Additionally, this comprehensive review included all types of spine surgery patients despite anatomical and biomechanical differences between the cervical and lumbar spine. Thus, future work in this area should (1) examine the type of spine surgery as a moderator of outcomes (i.e., do the effects of exercise-based programs depend on the type of spine surgery?); and (2) include both subjective and objective indices, as well as both acute postoperative outcomes (e.g., length of hospital stay, time to functional recovery, acute postoperative pain) *and* long-term outcomes (e.g., chronic post-surgical pain, re-operation, physical function, disability, quality of life, opioid use).

### Future Directions and Recommendations

Rigorous randomized trials are needed to test the existing and novel approaches aimed at promoting physical activity and pain outcomes in spine surgery patients. Current research indicates that post-surgical interventions may yield more robust long-term outcomes than preoperative interventions, although there are no studies directly comparing pre- vs. post-surgical approaches and this could be a focus of future trials. Alternatively, pre- and post-surgical programs could be studied as complementary interventions, facilitating physical activity and functioning throughout the perioperative period. Integrated approaches that include psychosocial intervention components may supplement exercise programs by addressing fear avoidance behaviors that interfere with engagement in activity, thereby maximizing short- and long-term benefits of exercise. Notably, efforts should be made to test brief and efficient programs that maximize accessibility for surgical patients. Some of the current programs are rather time-intensive, totaling nearly 40 h of intervention post-surgery. Such programs may not be feasible to implement across medical systems and surgical populations. Trials comparing the effects of different types of exercise (e.g., stretching, strengthening, endurance, aerobic, core stabilization) and timing of intervention (e.g., pre-surgery, immediately post-surgery, a few weeks after surgery) on short- and long-term post-surgical outcomes can inform the development of more efficient and effective protocols. Different types of exercise may differentially benefit patients depending on their background characteristics and the type of surgery, and this should be investigated. The use of a targeted population and approach, such as high-risk deconditioned patients and/or community-based training specific to a patient’s context, may also enhance acceptability, efficiency, and effectiveness.

Given the added value of postoperative psychological intervention on long-term disability and fear avoidance behavior, future studies should continue exploring ways to efficiently integrate such intervention into perioperative protocols. More recently, some physical therapy protocols (e.g., psychologically informed physical therapy, enhanced fear–avoidance rehabilitation) have begun incorporating elements of cognitive behavioral therapy, including addressing unhelpful cognitive patterns such as catastrophizing—reductions which may mediate some of the benefits of these treatments [55]. However, further research is needed given that a psychological intervention may optimize the efficacy of certain exercises but not others. Findings from meta-analyses suggest that postoperative psychotherapy may be more effective with exercises that reduce stress levels such as warm-up and aerobic exercise [40], but rigorous trials are needed to test these inferences. Researchers could also experiment with different types of therapeutic approaches. Though there is limited support for preoperative CBT, other psychological approaches may effectively enhance physical activity and pain outcomes in spine surgery patients. For example, acceptance and commitment therapy (ACT) has strong support for treating patients with chronic pain, including those with comorbid opioid use disorder [56,57,58,59,60]. More recently, ACT has been adapted for use in surgical patients and has been shown to reduce the amount of time with pain and on opioids post-surgery [61,62,63,64]. Although physical activity is not an explicit target of ACT, it may be a positive byproduct. A meta-analysis showed a significant small-to-moderate effect of ACT-based interventions on physical activity [65]. ACT may indirectly promote physical activity via acceptance. ACT uses a variety of acceptance-based metaphors and experiential exercises to increase awareness and acceptance of unwanted thoughts, feelings, and sensations, such as the emotional and/or physical discomfort that may be experienced during physical activity. Studies have indeed found that pain acceptance is related to improved physical functioning [66].

Finally, it is recommended that future studies test interventions focusing on other relevant behavioral and cognitive targets in addition to physical activity (e.g., social support, pain catastrophizing, pain self-efficacy) to effectively enhance recovery and long-term outcomes after spine surgery. For example, recent studies have suggested that reductions in catastrophizing and fear of movement mediate the beneficial effects of CBT on improving pain and function after a variety of surgeries, including knee surgery [67] and spine surgery [68]. Interventions targeted at such crucial psychosocial process variables and delivered in “high-risk” samples of patients (e.g., those who are sedentary or high in catastrophizing and fear of movement) may optimize the beneficial outcomes of such treatment approaches.

## Data Availability

No new data were created or analyzed in this study. Data sharing is not applicable to this article.
